# Unveiling the Uncommon: A Case of Metastatic Ewing Sarcoma of the Kidney

**DOI:** 10.7759/cureus.52970

**Published:** 2024-01-26

**Authors:** Ahmed D Khudair, Aiman D Khudair, Thuraiya Al-Rawahia, Rachel A Marshall, Khalifa Albenjasim, Mahera Roohi, Ziad Al Naib

**Affiliations:** 1 Department of Urology, Royal College of Surgeons in Ireland - Bahrain, Busaiteen, BHR; 2 Department of Urology, King Hamad University Hospital, Busaiteen, BHR; 3 Department of Pathology, King Hamad University Hospital, Busaiteen, BHR

**Keywords:** ewing sarcoma of the kidney, chemotherapy, metastasis, extraosseous ewing sarcoma, ewing sarcoma

## Abstract

Primary Ewing sarcoma of the kidney (ESK) is a rare and aggressive entity, with a poor prognosis. It often presents as metastatic disease with the lungs being the most common site. In adults, the occurrence of these tumors is uncommon, with patients exhibiting non-specific symptoms such as weight loss, flank pain, hematuria, and an abdominal mass. The combination of these vague clinical symptoms and the rarity of these tumors often results in a delayed diagnosis, leading to poorer outcomes for these patients. We present a case of a 38-year-old female with metastatic ESK. The patient initially presented with abdominal pain, vomiting, and a four-day history of constipation. The diagnosis was confirmed through computed tomography scans, ultrasound-guided biopsy of the lesion, and fluorescence in situ hybridization that revealed translocation of the *EWS* gene on chromosome 22q12. She was managed with chemotherapy regimens and palliative care; however, the disease progressed and she passed away six months after her initial diagnosis.

## Introduction

Ewing sarcoma is a poorly differentiated and highly malignant tumor that usually arises in bone and soft tissue [1,2]. The presence of small round blue cells is characteristic of this tumor as well as the rearrangement of the *ESWR1* gene on chromosome 22 [3]. It is most commonly diagnosed in young adults and adolescents with a peak incidence at 15 years of age [1]. The occurrence of Ewing sarcoma of the kidney (ESK) is rare, comprising only 1% of all renal tumors [4]. Patients generally present with a mass in the abdomen or with renal colic symptoms like abdominal pain and hematuria [4]. Subsequently, due to its occult intra-abdominal location, the tumor usually presents as a sizeable mass before it is detected [2]. Given the aggressive nature of this tumor, metastasis occurs in 65% of cases, frequently involving the lungs but can also spread to regional lymph nodes and the liver [4].

Diagnosis can be achieved through radiological modalities such as magnetic resonance imaging (MRI) and computed tomography (CT) scans which help identify tumor size, location, and extent of distant or local metastasis [3]. Histopathology coupled with immunohistochemistry (IHC) and cytogenic studies are also vital to make a definitive diagnosis of Ewing sarcoma [5]. Treatments include a multimodal approach encompassing surgical resection and/or radiotherapy [2]. Additionally, adjuvant chemotherapy is administered using a combination of vincristine, doxorubicin, and cyclophosphamide (VDC) followed by ifosamide and etoposide (IE) [6]. However, ESK is associated with a poor prognosis with a survival of less than one year [7]. The poor prognosis is attributed to factors such as delayed diagnosis, large size, aggressive nature, and high risk of metastasis [3]. 

In this case report, we present a 38-year-old female patient with metastatic ESK.

## Case presentation

A 38-year-old female presented to the emergency department complaining of a one-week history of diffuse abdominal pain primarily in the left lumbar region that worsened over the past two days. She also mentioned a four-day history of constipation and two episodes of non-bilious vomiting on the day of the presentation. During the past six months, she had experienced progressive abdominal distension with intermittent episodes of constipation. Her prior medical and surgical history was significant for morbid obesity and two cesarean sections. She had no significant prior family history. On physical examination, her abdomen was soft and distended with a palpable abdominal mass. Her laboratory investigations revealed an elevated lactate dehydrogenase of 1388 U/L (normal range: 100-190), an anemic blood picture with a hemoglobin of 8.5 g/dl (normal range: 12-16), elevated C-reactive protein of 80.7 mg/L (normal range: 0-10), and low serum chloride of 98 mmol/L (normal range: 100-108).

**Figure 1:**
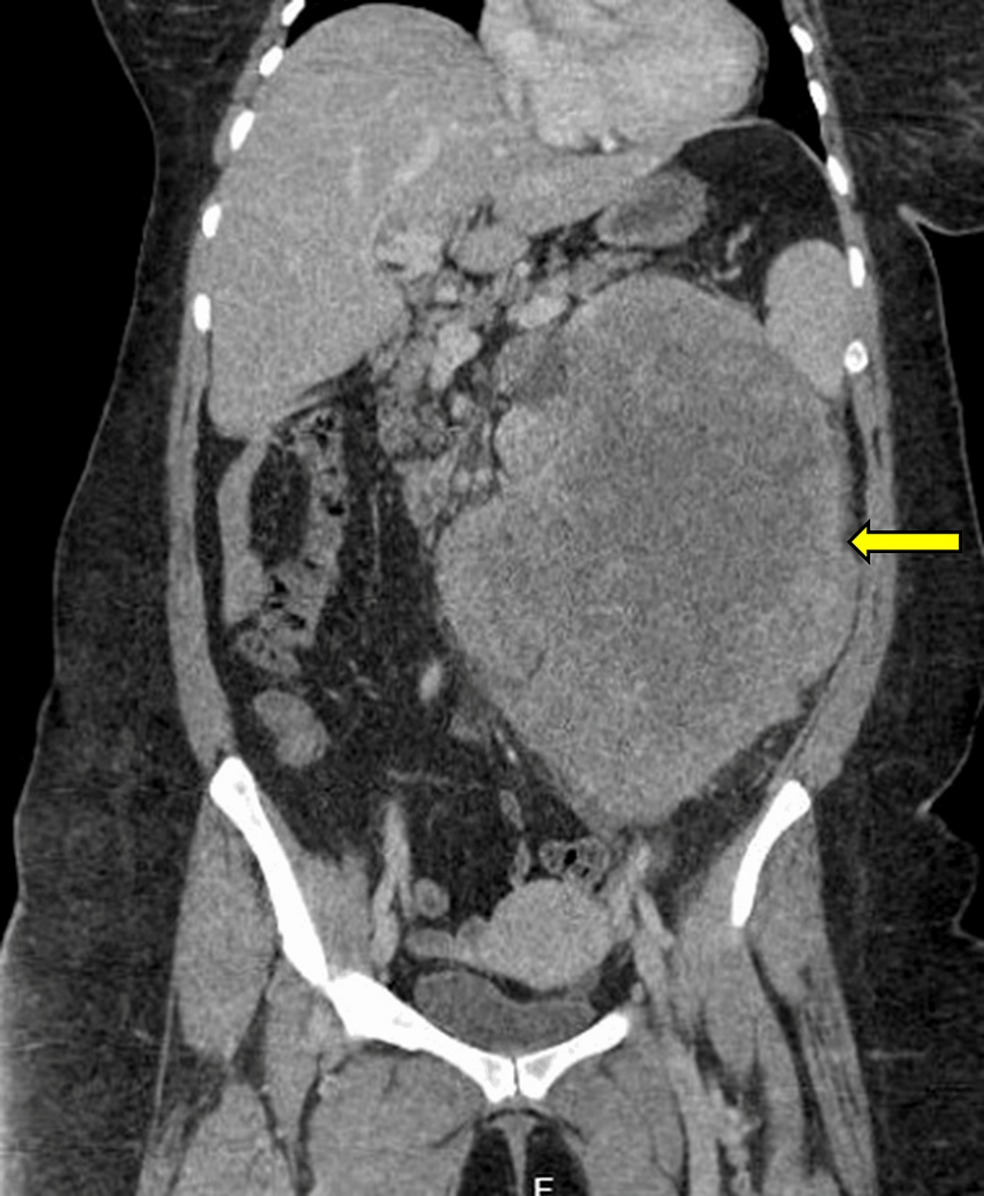
Coronal CT image of primary Ewing sarcoma of the left kidney measuring 25 cm x 18 cm x 18 cm (arrow) CT: computed tomography

**Figure 2:**
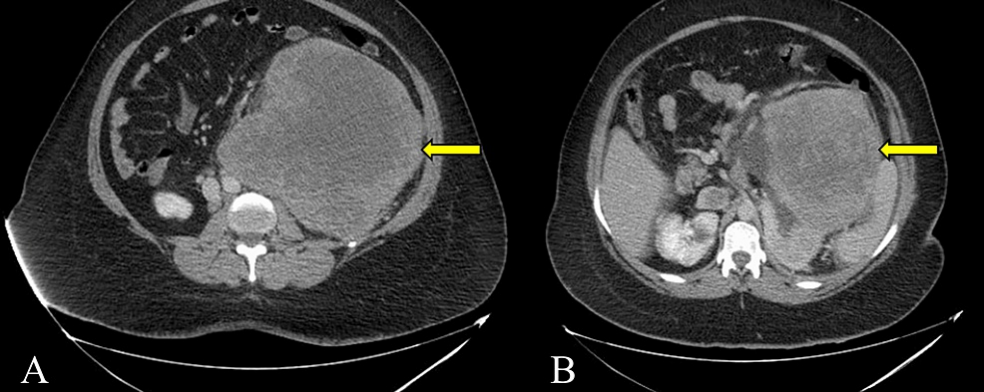
Axial CT image of primary Ewing sarcoma of the left kidney measuring 25 cm x 18 cm x 18 cm (arrows) CT: computed tomography

A CT scan was performed, which showed a large heterogenous non-calcific left renal exophytic mass measuring approximately 25 x 18 x 18 cm (Figures 1, 2). Retroperitoneal adenopathies and multiple bilateral pulmonary nodules were also noted with the largest located in the posterior basal segment of the right lower lobe measuring 32 x 31 mm. This raised suspicion of a left renal cell carcinoma (RCC) prompting further workup.

**Figure 3:**
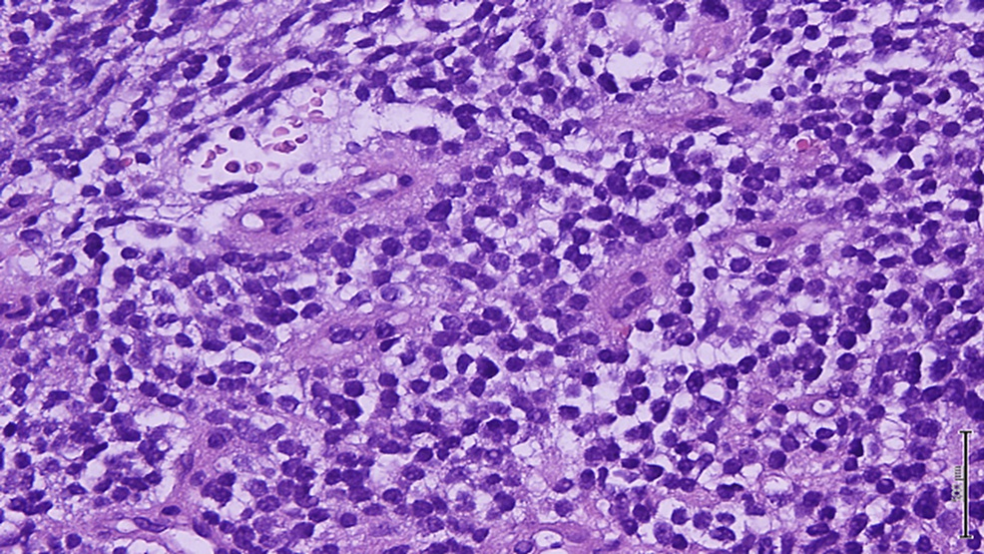
H&E (20x) sections showing sheet-like growth pattern, uniform small round cells, finely stippled chromatin, inconspicuous nucleoli, and scant clear to eosinophilic cytoplasm with indistinct cytoplasmic membranes H&E: Hematoxylin and eosin

**Figure 4:**
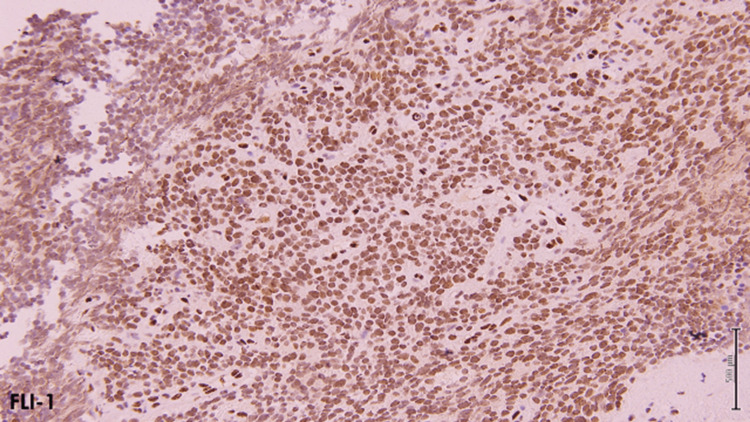
FLI-1 immunohistochemical stain (10x) demonstrates strong nuclear positivity representing a surrogate marker for characteristic EWSR-FLI-1 translocation FLI-1: Friend leukemia integration 1; EWSR: Ewing sarcoma breakpoint gene

**Figure 5:**
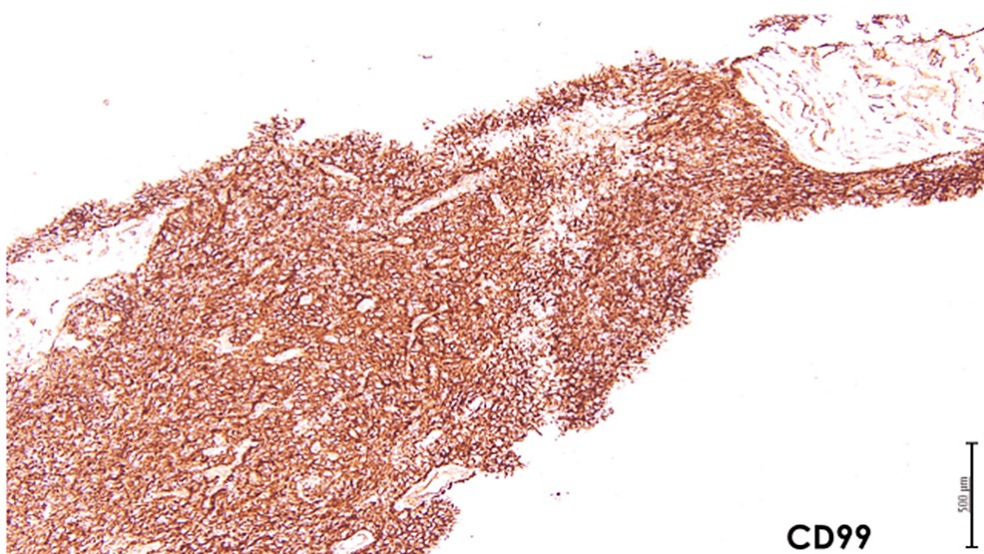
CD99 immunohistochemical stain (4x) shows strong diffuse membranous positivity in the tumor cells

An ultrasound-guided biopsy of the left renal mass was performed and histopathological analysis revealed a small round blue cell tumor with a densely sclerosed stroma and marked necrosis. A repeat biopsy was requested as the preserved tissue was too scant for a definitive diagnosis. A subsequent biopsy of the left renal mass was performed which showed cores of a malignant neoplasm, composed of sheets of round blue cells, with a high nucleocytoplasmic ratio, round nuclei, and focal areas of necrosis (Figure 3). IHC staining showed positivity of CD99 and FLI-1, a focal weak positivity of synaptophysin, and an intact INI-1 (Figures 4, 5). Desmin, myogenin, pancytokeratin, and WT-1 were negative. Fluorescence in situ hybridization (FISH) was done and showed *EWSR1* gene translocation at 11:22. The final diagnosis was a small round blue cell tumor consistent with Ewing sarcoma.

She was started on the VDC protocol and sodium 2-mercaptoethane sulfonate, and then treated with IE. During her treatment, she suffered progressive peripheral neuropathy, which was managed with intravenous immunoglobulin, likely due to a paraneoplastic syndrome. Unfortunately, the patient suffered cardiac arrest in the intensive care unit and succumbed to her complications six months after her initial presentation.

## Discussion

Ewing sarcoma is a cancer of bone and soft tissue [1]. It is thought that the cells of origin for this disease arise from neural crest cells or mesenchymal stem cells [8]. The majority of cases of Ewing sarcoma occur in bone with the most frequent sites being the lower extremity and pelvis, while extraosseous sites account for 20-30% of patients [9,10]. Metastases commonly occur in the lungs, bone, and bone marrow [11]. ESK is an exceedingly rare and highly malignant entity, first reported by Seemayer et al. in 1975 and approximately comprising less than 200 cases in the literature [12-14]. These tumors, which make up <1% of all renal tumors, have a much higher prevalence in males than females [4]. A meta-analysis done by Risi et al. of 116 cases of ESK found that the median age was 28 years old and the most common presenting clinical symptoms included pain (54%), hematuria (29%), and renal mass (28%) [15]. In our case, our patient was a 38-year-old female who presented with diffuse abdominal pain localizing in the left lumbar region, a four-day history of constipation, and two episodes of non-bilious vomiting. 

ESK frequently spreads to various locations in approximately 65% of patients, with the lungs, liver, and regional lymph nodes being the most commonly affected sites [4]. Our patient had metastases to the lungs. ESK has a grim outlook, as 66% of patients are diagnosed with stage IV disease at presentation, and those with metastases typically have a median overall survival of 24 months [15]. In our case, our patient presented late with metastases, which conferred a poor prognosis and she passed away six months later. This highlights the highly aggressive nature of ESK.

Imaging should not be used as the primary diagnostic tool for ESK since it has no specific imaging features and could be potentially misdiagnosed as RCC [16]. Given the inherent vagueness of imaging, greater emphasis should be placed on histopathology, IHC, and cytogenic studies to confirm the diagnosis of ESK. A renal mass biopsy is performed to obtain tissue samples for histological analysis. Although the risks of biopsy for renal mass are rare, they are not insignificant. Hematoma and notable pain are among the most common complications [17]. Other risks include infection, arteriovenous fistula, pneumothorax, hemorrhage, and tumor seeding along the needle tract [18]. In our case, the biopsy was performed due to suspicion of RCC.

On histology, it is classically characterized by the presence of small round blue cells that exhibit a Homer-Wright rosette arrangement [19]. Immunohistochemically, expression of cell surface antigen FLI-1 and CD99 is present in 60.6% and 99% of ESK cases, respectively. In the present case, IHC was positive for both CD99 and FLI-1. Furthermore, this disease is characterized by the t(11;22)(q24;q12) translocation found in 85% to 95% of all patients affected [5]. This translocates the *EWSR1 *on chromosome 22 to the *FLI1* gene on chromosome 11 [5]. In our case, the *EWSR1* translocation at 11:22 was detected by FISH. 

To date, there is a lack of consensus regarding the most effective treatment of ESK. However, the standard treatment involves a multimodal approach consisting of surgical resection, chemotherapy, and radiotherapy. The traditional chemotherapy treatment consists of alternating between the VDC and the IE regimens. Alternating between both drug combinations has been shown to significantly improve the outcome in non-metastatic Ewing sarcoma [20]. However, this regimen is associated with various hematological and non-hematological toxicities. Grade 3 and 4 hematological toxicities are common, encompassing anemia, thrombocytopenia, and neutropenia [21]. Additional common grade 3 and 4 non-hematological toxicities include bacteremia, urinary tract infections, mucositis, and fever [21]. Moreover, the prognosis of patients with metastatic ESK is poor despite the aggressive treatment with a cure rate of 20% [16].

## Conclusions

ESK is a rare yet highly aggressive tumor. Our case emphasizes the importance of considering it in the differential diagnoses of aggressive renal masses in the adult population. The definitive diagnosis is strongly based on histopathological findings, IHC, and cytogenetic studies. Despite the treatment options, ESK is known to have a poor overall survival rate. Therefore, greater emphasis must be placed on early accurate diagnosis and timely treatment in order to manage this highly aggressive disease.
